# Genetics, early-life factors and brain imaging in children with autism spectrum disorder

**DOI:** 10.1192/j.eurpsy.2026.10553

**Published:** 2026-07-31

**Authors:** P. Querol Clares, M. Alijotas Capdevila, M. Martinez Ramirez, I. Setien Ramos, J. Lugo Marin, A. G. Lungo Peccorini, L. Gisbert Gustemps, J. A. Ramos Quiroga

**Affiliations:** 1 Psychiatry; 2 Pediatric Neurology, Hospital Vall d’Hebron, Barcelona, Spain

## Abstract

**Introduction:**

Autism spectrum disorder (ASD) is a neurodevelopmental disorder influenced by common polygenic variation, rare high-impact genetic variants, and non-genetic factors. Gene–environment interactions are increasingly recognized as potentially relevant, although their precise role in ASD heterogeneity and variable expression remains incompletely understood. However, current research seldom provides a joint description of genetic, early-life factors, and clinical features, highlighting the need for more comprehensive studies.

**Objectives:**

To analyze prenatal, perinatal, and postnatal factors, along with clinical characteristics and findings from complementary assessments in a cohort of children with ASD.

**Methods:**

A cross-sectional study was conducted at a Child and Adolescent ASD unit in a tertiary hospital. Inclusion criteria were a DSM-5 diagnosis of ASD and age up to 18 years. The exclusion criterion was insufficient information according to the study objectives. Of the 260 patients under follow-up, 224 were included in the analysis. Data collection encompassed prenatal, perinatal, and postnatal factors, clinical characteristics and findings from complementary investigations.

**Results:**

The study sample comprised 78% male and 22% female patients. The mean age was 9.8 years, with the majority falling between 5 and 10 years (48%), and the remainder distributed as 22% aged 11–15, 16% >15, and 14% <5 years. Regarding ASD severity, 53% of patients were classified as grade 1, 30% as grade 2, and 17% as grade 3. In addition, 41% had intellectual disability (mild:65%, moderate:23%, severe:12%) while 80% showed early childhood language impairments.

Concerning prenatal factors, mean maternal and paternal ages were 34 and 36 years, with most parents in the 30–39 year range (67% and 58%, respectively). Attention-deficit/hyperactivity disorder was the most frequently reported familial comorbidity, present in 86% of cases. Perinatal factors included cesarean delivery(31%), prematurity(21%) and eclampsia/preeclampsia(7%). Postnatal data showed a mean birth weight of 2870 g, with 22% classified as low birth weight (<2500 g).

Complementary investigations revealed pathological findings in 11% of genomic arrays, 9% of exome analyses and abnormal brain MRI in 86% of patients. Genetic testing predominantly yielded non-diagnostic results or variants of uncertain significance, with a higher pathogenic yield from copy number variants than from exome sequencing. ASD-relevant findings included NRXN1 deletions, SHANK3 variants, 16p11.2 duplications, 22q11.2 rearrangements and sex chromosome aneuploidies.

**Conclusions:**

This cross-sectional study underscores that ASD is frequently associated with early-life complications, language impairments and neuroimaging abnormalities, while genetic testing yields both pathogenic and inconclusive findings. These findings reinforce the need for integrative approaches to advance precision in ASD.

**Disclosure of Interest:**

None Declared

